# International best practice in the cessation of vaping and non**-**combustible nicotine use, and barriers, facilitators and treatment preferences: A rapid review

**DOI:** 10.18332/tpc/226396

**Published:** 2026-10-16

**Authors:** Rory Coyne, Mengyang Zhang, Linda van der Spek, Rishi Tadiparti, Gintare Valentelyte, Paul Kavanagh, Ken Ward, Zubair Kabir, Brian Doyle, Jan Sorensen, Frank Doyle

**Affiliations:** 1 Department of Health Psychology School of Population Health, RCSI University of Medicine and Health Sciences Dublin Ireland; 2 Division of Neonatology, Department of Neonatal and Paediatric Intensive Care Erasmus MC University Medical Centre Rotterdam the Netherlands; 3 School of Medicine, RCSI University of Medicine and Health Sciences Dublin Ireland; 4 Centre for Chronic Diseases and Population Health Research (Converge), School of Population Health, RCSI University of Medicine and Health Sciences Dublin Ireland; 5 Department of Public Health and Epidemiology School of Population Health, RCSI University of Medicine and Health Sciences Dublin Ireland; 6 HSE Tobacco Free Ireland Dublin Ireland; 7 College of Population Health, The University of New Mexico Albuquerque New Mexico; 8 School of Public Health, University College Cork Cork Ireland; 9 Healthcare Outcomes Research Centre, School of Population Health, RCSI University of Medicine and Health Sciences Dublin Ireland

**Keywords:** nicotine addiction, e-cigarettes, vaping, best practice, implementation factors

## Abstract

**Introduction:**

Increasing use of e-cigarettes and forms of non-combustible nicotine consumption, especially among children and young people who do not smoke, is an emerging challenge for traditional services oriented to stop smoking care. However, as services struggle to manage these newer presentations, there is a paucity of guidance for supporting cessation. This rapid review sought to evaluate the extent to which interventions for the cessation of vaping and non-combustible nicotine products align with best practice, and to understand what barriers, facilitators and user-reported preferences for service features have been reported.

**Methods:**

A systematic search of four electronic databases (MEDLINE, PsycINFO, PsycEXTRA, and Scopus) was conducted on 18 November 2025 to search for vaping and non-combustible nicotine cessation interventions published since 2003. The included interventions were evaluated using a framework to assess the extent to which they aligned with best practice in public health. Information regarding barriers and facilitators to implementation, as well as user-reported preferences for intervention delivery, were extracted and synthesized in a narrative synthesis.

**Results:**

Findings from 23 studies (n=10373) revealed that alignment with best practice was highest in the areas of relevance (16/23), replicability (14/23) and effectiveness (12/23). Alignment was poorest in the areas of stakeholder collaboration (3/23) and sustainability (3/23). Interventions which prioritized accessibility, flexibility, and autonomy were noted by authors as facilitators to implementation. Participants demonstrated a preference for digital, low-burden interventions with social support.

**Conclusions:**

This rapid review identified opportunities to more effectively embed best practice principles in intervention design and delivery. The findings of this review can be used to inform the development of services which aim to reduce the impact of nicotine addiction fueled by vaping and non-combustible nicotine product use.

## Introduction

### Background

Tobacco use presents a global public health challenge. In response to this, and in line with World Health Organization (WHO) measures to reduce tobacco use globally, many countries have established stop smoking services, the evidence base for which is well-established^1^. The use of vapes, also commonly referred to as ‘e-cigarettes’, has recently seen a sharp increase in uptake which both threatens to undo decades of progress made by tobacco cessation initiatives, and introduce the highly addictive chemical nicotine to individuals who would previously not have interacted with it. In addition, the number of alternative nicotine products available on the market has also grown in recent years. These forms of nicotine consumption do not rely on combustion for nicotine delivery, and include products such as heated tobacco (or ‘heat-not-burn’), oral nicotine pouches, and smokeless tobacco (such as snus). Young adults, amongst whom vape use has increased nine-fold from 2021 to 2023, are particularly vulnerable, both to developing a nicotine dependence and progressing to smoking^2,3^. This presents a dilemma for services that have traditionally delivered smoking cessation supports, and need to respond to this growing challenge.

Across many countries, there is currently a lack of dedicated cessation services to support users of vapes and non-combustible nicotine products who want to quit. Evidence suggests that the concurrent use of vapes alongside non-combustible forms of nicotine is currently low, but is steadily rising^4^. Individuals have a variety of motivations for using these products, including as a smoking cessation aid. At the same time, those who are motivated to quit, and thus become nicotine-free, may struggle to do so without readily available, dedicated cessation services. In its recommendations for treatment approaches to tobacco cessation, the WHO found moderate-to-low certainty for the use of behavioral support and pharmacological approaches designed to help users of non-combustible nicotine products quit^1^. The use of products such as snus, snuff and dip, predate the proliferation of vaping; however, both types of products deliver nicotine without the combustion of tobacco, thus diverging distinctly from traditional smoking in similar ways. Furthermore, the use of vapes in tandem with non-combustible nicotine products is rising slowly over time in regions such as England^4^. Qualitative evidence also suggests that individuals are using products such as nicotine pouches (which deliver synthetic nicotine orally through a small sachet placed in the mouth) as a substitution for other products^5^. It could therefore be of significant value to consider both types of products in tandem by adopting a broader nicotine dependence perspective, rather than considering vaping in isolation.

The rise of novel nicotine products requires standardized, best-practice guidelines to aid clinicians in supporting those who want to quit. Ng and De Colombani^6^ developed a framework for identifying best practices in public health interventions. The framework provides eight clear criteria for evaluating alignment with best practice among public health interventions – relevance, community participation, stakeholder collaboration, ethical soundness (defined by the authors as ensuring equitable distribution of resources, considering vulnerable groups, and demonstrating respect for local norms), replicability, effectiveness, efficiency, and sustainability. The framework provides examples for how each criterion may be evidenced in various ways – for instance, an intervention may demonstrate relevance by evaluating disease burden in the community, or involving target groups and stakeholders in a needs assessment. The use of a framework to identify best practice is consistent with international public health policy approaches. The Organization for Economic Cooperation and Development (OECD) Guidebook on Best Practices in Public Health recommends that best practice be identified based on multiple criteria^7^. The framework by Ng and De Colombani^6^ was cited as an example. Therefore, it provides a structured multi-criteria assessment framework which is consistent with international public health best practice evaluation approaches.

Many vape users face significant barriers to engaging with cessation programs, such as fear of relapsing to smoking, particularly when the precise health consequences of nicotine delivered in these novel forms are still largely unclear. While what is currently known primarily relates to motivational influences, barriers to implementation also exist, and this has been underexplored to date. The extent to which an intervention is implemented successfully has a sizeable influence on engagement. Sustained engagement with interventions is also associated with the success of smoking cessation interventions^8^. Relatedly, supporting engagement by tailoring interventions to users’ preferences could also maximize the effectiveness of vaping and non-combustible nicotine product cessation programs. Engagement is a multifaceted construct, and requires taking a broader view by examining user-reported preferred modes of delivery, intervention duration, and intervention content, to understand of how best to support nicotine cessation.

### Aims of this study

This rapid review aimed to assess the extent to which interventions for vaping and non-combustible nicotine product cessation align with best practice. In doing so, it sought to make a distinct contribution to the literature over previous reviews which have simply evaluated the effectiveness of these interventions. The second aim of this review was to synthesize the barriers and facilitators that are affecting the implementation of these interventions. The third aim was to synthesize the user-reported treatment preferences for intervention features, which can have a sizeable bearing on sustained engagement and adherence. This review focused on interventions targeting both vaping and non-combustible nicotine products. This is both of practical value and is in step with an increasingly poly-nicotine landscape, whereby users frequently switch or combine products.

Therefore, this rapid review sought to address the following research questions: 1) ‘Which approaches to the management and cessation of vaping and/or non-combustible nicotine product use can be considered best practice?’; 2) ‘What are the barriers and facilitators to the implementation of interventions for vaping cessation and/or non-combustible nicotine product use?’; and 3) ‘What treatment preferences have been reported by those who vape and/or use non-combustible nicotine products?’.

## Methods

The protocol for this rapid review was submitted for registration to the International Prospective Register of Systematic Reviews (PROSPERO) on 8 September 2025 (ID: CRD420251141677). A PRISMA 2020 checklist for this review was published on the Open Science Framework: 10.17605/OSF.IO/5 × 9R6. The review was undertaken as a rapid review, which uses systematic review methods in a streamlined manner to expedite the evidence synthesis process. A rapid review approach was chosen as an effective means of provide timely, decision-relevant evidence whilst maintaining methodological rigor. Rapid reviews, rather than being a distinct methodology, seek to modify one or more steps of the systematic review process. Hence, we limited our search to three databases of peer-reviewed journal articles, plus one database of grey literature, and deliberately omitted performing a critical appraisal of the included studies. The eligibility criteria for the review were developed using the Patient, Intervention, Comparator, Outcome(s), Study (PICOS) framework, and are reported below.

### Selection criteria

An overview of the eligibility criteria is provided in Table 1. As identified in a Cochrane review by Butler et al.^9^, interventions can include behavioral or pharmacological approaches, as well as changes in the characteristics of products used – or a combination of approaches. We included individuals who were motivated to quit vaping and/or non-combustible nicotine products, operationalized as volunteering to participate in a cessation intervention or attending a cessation service. Studies which recruited participants who were using these products exclusively as a smoking cessation aid or on the advice of a healthcare practitioner at the time of the study were excluded. Participants who used traditional cigarettes in tandem with vapes and/or non-combustible nicotine products were still included, but participants who used traditional cigarettes only were excluded. ‘Intervention’ refers to individual and/or group-level support for the cessation of vaping and/or non-combustible nicotine products, provided to those who are motivated to quit. Studies that used either quantitative, qualitative, or mixed methods were included. Grey literature (conference proceedings, preprints, etc.) were also considered for inclusion.

**Table 1:** Summary of eligibility criteria used in this review, used to identify 23 vaping and non-combustible nicotine cessation interventions, using a systematic search of three databases of peer-reviewed journal articles (MEDLINE, PsycINFO, Scopus), published between 1 January 2003 and 18 November 2025. Eligibility criteria were based on the PICOS framework

	Include	Exclude
**Population**	Participants who use vapes and/or non-combustible nicotine products, and were motivated to quit by virtue of signing up to a cessation intervention.	Participants who used vapes and/or non-combustible nicotine products exclusively as a smoking cessation aid, or on the advice of a healthcare practitioner; participants who used cigarettes only.
**Intervention**	Interventions of any type which target vaping and/or non-combustible nicotine use.	Does not explicitly target vaping or non-combustible nicotine use; does not concentrate on cessation of these products.
**Comparator**	Study selection was not guided by the use of comparators.	
**Outcome**	Collects outcome data on the cessation or use of vapes and/or non-combustible nicotine products.	Does not collect data on the cessation or use of vapes or non-combustible nicotine products.
**Study**	Peer-reviewed journal articles and grey literature on primary research using qualitative or quantitative methods published between 2003 and 2025 in the English language.	Studies not published in English; studies published before 2003; review articles.

### Search strategy

We searched three electronic databases of peer-reviewed journal articles (MEDLINE, PsycINFO and Scopus) and one electronic database of grey literature (PsycEXTRA) for records on 18 November 2025. The search strategies for each database (which can be found in Supplementary file Appendix A) comprised three concept blocks. The first block of terms related to products, such as ‘vapes’, ‘electronic nicotine delivery system’, and ‘oral nicotine’. The second block related to intervention types, using terms such as ‘cessation’, ‘quitline’, and ‘trial’. The third and final block centered around barriers, facilitators or treatment preferences, and used a broad set of terms including ‘acceptance’, ‘feasibility’, and ‘engagement’. Terms within each concept block were combined using the OR Boolean operator, and the three concept blocks were then combined with the AND operator. Each database was then queried using their respective date (1 January 2003 until 18 November 2025) and language (English only) filters. The full search strategy for each database can be found in Supplementary file Appendix A.

### Screening and data extraction

Once the search had been completed, the results were exported from the respective databases to Zenodo, at which point the references were imported into Covidence. Covidence’s automatic de-duplication method was used, the outcome of which was checked by one reviewer (RC) for accuracy. Screening was then conducted within Covidence in two stages: 1) title and abstract screening, and 2) full-text screening of articles which were deemed eligible at stage 1. All screening was conducted by one independent reviewer (RC), with an additional 10% screened by two independent reviewers (LvdS, RT) meaning that 20% of articles were double screened, in line with best practice guidelines for screening^10^. Disagreements were handled through a majority vote among the three reviewers.

We extracted the following information from the final sample of included studies: 1) Study characteristics (authors, year, journal, country, setting, and eligibility criteria); 2) Demographic information (sample size, age, gender); 3) Types of product(s) used; 4) Frequency of product usage (i.e. daily, weekly, past 30 days, etc); 5) Intervention characteristics (intervention content, frequency of intervention, modality of intervention, duration of intervention; 6) Barriers; 7) Facilitators; 8) Treatment preferences (defined in this review as quantitatively or qualitatively explored user responses to included interventions); and 9) Data on intervention effectiveness (cessation rates, changes in product use frequency, etc). Data extraction was conducted by three independent reviewers (RC, LvdS, RT). A data extraction table was used to compile the extracted data, and adapted for publication into a table showing the characteristics of included studies (Supplementary file Appendix B).

### Data synthesis and analysis

To evaluate the extent to which interventions aligned with best practice, a framework devised by Ng and De Colombani^6^ was used. Included interventions were assessed according to the eight criteria provided by this framework in a binary, yes/no manner. Examples from the article were provided in a scoring sheet to justify where an intervention was deemed to have fulfilled a given criterion. All assessments were conducted by two independent reviewers (RC, LvdS). To meet the remaining objectives of the review (synthesizing the evidence for barriers, facilitators, and treatment preferences), a narrative synthesis, in which the results are presented textually to generate a common set of themes across studies, was conducted.

Barriers and facilitators were predominantly identified and extracted from included studies in the form of author-reported factors which helped or hindered intervention implementation, and were mainly found in the strengths and limitations portion of the article. Barriers were therefore defined as any factor related to the study’s methodology which was deemed to have hindered or impeded successful implementation of the intervention. Facilitators, on the other hand, were defined as any factor related to the study’s methodology which was deemed to have supported or enabled successful implementation. Treatment preferences were presented as either quantitative measures of acceptability or perceived efficacy of the intervention, or qualitative data on experiences or attitudes towards the intervention. Barriers and facilitators are presented in tandem to facilitate a richer narrative synthesis, as a common set of factors were identified across included studies.

## Results

The initial database search on 18 November 2025 yielded a total of 5010 results. The PRISMA flow diagram for this review can be found in Figure 1. Once duplicates were removed the titles and abstracts of 3854 references were screened for eligibility, of which 3751 were excluded. The remaining 103 full-text articles were then assessed, and a further 80 articles were excluded. This resulted in a final sample of 23 studies obtained from the database search and included in the review. Twenty-two primary studies^11-31^ were included, with Graham et al.^18^ being a secondary data analysis of Graham et al.^17^, included as it contained data relevant to the review.

**Figure 1:**
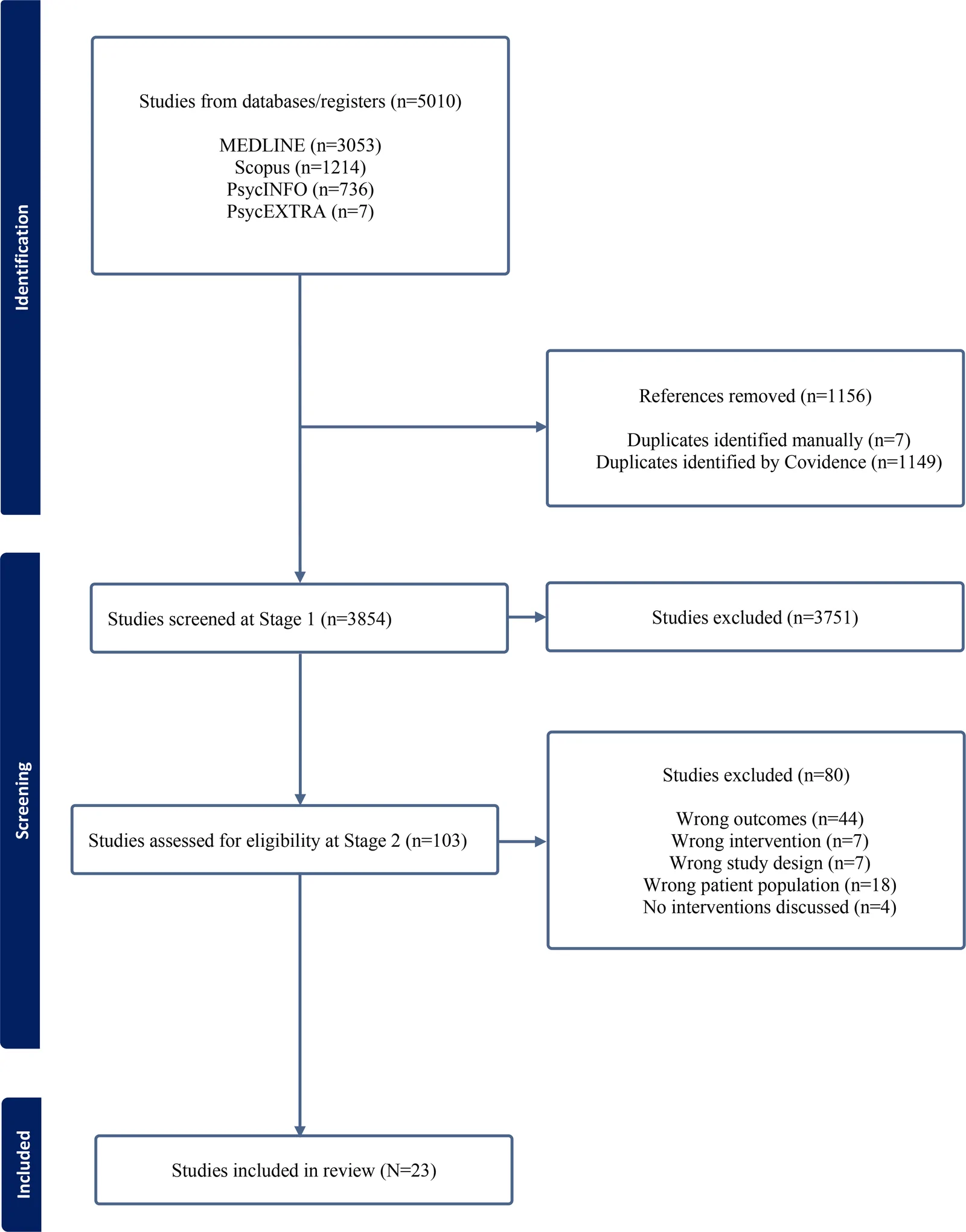
PRISMA flow diagram overviewing the searching and screening processes used to identify 23 vaping and non-combustible nicotine cessation interventions, using a systematic search of three databases of peer-reviewed journal articles (MEDLINE, PsycINFO, Scopus), published between 1 January 2003 and 18 November 2025

### Study characteristics

A full summary of study characteristics can be found in Supplementary file Appendix B. Some general characteristics of the included studies are provided below.

#### Population

The 23 studies recruited a total of 10373 participants. The majority of studies were conducted in the United States^11,13-15,^^17,18,20,22-26,28,31-33^ (n=16; 69.6%), with studies also including participants from Italy (1)^12^, Canada (1)^13^, Norway (1)^16^, Sweden (1)^16^, Pakistan (3)^27,29,30^, Bangladesh (1)^29^, Switzerland (1)^19^, and the United Kingdom (3)^27,29,30^ (several studies recruited participants across multiple countries). In terms of product users, 14 (60.87%) recruited vape users ^12,15,17-20,^^22,24-26,28,31-33^, and the remaining 9 (39.13%) recruited users of non-combustible nicotine^11,13,14,16,21,23,27,30^ – where product type was stated, individuals used snus (1)^16^, chewable tobacco (2), snuff, (2)^11,23^ and dip (1)^27^. No studies were identified which concentrated on novel, synthetic nicotine products, such as nicotine pouches.

#### Design

A combination of non-randomized (n=17), and randomized (n=6)^11,15,16,20,28,29^ intervention studies were identified. In terms of the modality of the intervention, the majority were delivered remotely, with text message-based programs (n=10)^17-20,22,24,25,32,33^ and internet or app-based programs (n=3)^13,25,31^ also used. The main types of in-person delivery methods used were face-to-face counselling (n=4) and over-the-counter pharmacological approaches which were delivered by clinicians or research staff (n=7)^12,14,16,21,28,29,31^ or mailed to participants (n=1)^14^.

Almost all interventions included some form of behavioral counselling, with 8 studies (as above) employing pharmacological intervention for vaping or nicotine cessation, and 2 studies^25,26^ used a financial incentive-based intervention (i.e. offering a financial reward, e.g. a shopping voucher, for quitting and staying nicotine-free). Among the eight studies which provided pharmacological support, this was mainly offered in the form of varenicline at between 0.5 and 4 mg^12,15,16,21^, nicotine replacement therapy at between 2 and 4 mg (which included lozenges^14^, gum^29^, and gum and/or patches^31^, and finally cytisinicline at 3 mg^28^.

#### Outcomes

Included interventions largely used a common set of outcome measures to evaluate effectiveness: point prevalence abstinence (PPA), continuous abstinence rates (CAR), relapse and lapse-related outcomes, and number of quit attempts made. The most commonly reported outcomes were PPA proportions, captured between 7 and 30 days – these were typically self-reported, but three studies used biochemical verification (e.g. cotinine^20^. The second most common outcomes were CAR, which were particularly common in pharmacotherapy studies. These outcomes evaluated continued abstinence over multiple defined intervals, such as between weeks 4–12 and weeks^12^ to evaluate short- and long-term abstinence. Eight studies evaluated prolonged abstinence by surveying participants over long-term follow-up, e.g. Boyle et al.^11^. The other two main types of outcome measures were assessments of relapse and lapse-related outcomes^28^ and the number of quit attempts made over a given period^13^.

### Which approaches can be considered best practice, according to a framework for public health interventions?

An overview of the findings concerning the extent to which included interventions were in alignment with best practice, evaluated by screening studies using the framework developed by Ng and De Colombani^6^, is presented in Table 2. Overall, the main areas in which included interventions demonstrated the greatest alignment with best practice were: relevance (16/23), replicability (14/23), and effectiveness (12/23). The areas in which alignment with best practice was poorest were: ethically sound (4/23), stakeholder collaboration, and sustainability (both 3/23). Where each of the eight criteria were demonstrated across the reviewed studies, this was evidenced in the following ways (accompanied by the percentage of included studies which evidenced the relevant criterion).

**Table 2:** Overview of evaluation of 23 included vaping and non-combustible nicotine cessation interventions, identified through a systematic search of three databases of peer-reviewed journal articles (MEDLINE, PsycINFO, Scopus), published between 1 January 2003 and 18 November 2025, in terms of their alignment with best practice^6^

Study	1 Relevance	2 Community engagement	3 Stakeholder collaboration	4 Ethically sound	5 Replicable	6 Effective	7 Efficient	8 Sustainable
Boyle et al.^11^	Yes	No	No	No	Yes	Yes	No	No
Caponnetto et al.^12^	No	No	No	No	Yes	Yes	No	No
Danaher et al.^13^	Yes	No	No	No	Yes	No	No	No
Do et al.^32^	Yes	No	No	No	Yes	No	No	No
Ebbert et al.^14^	Yes	No	No	No	Yes	No	No	No
Evins et al.^15^	Yes	No	No	No	Yes	Yes	No	No
Fagerstrom et al.^16^	Yes	No	No	No	Yes	Yes	No	No
Graham et al.^33^	Yes	Yes	No	No	Yes	Yes	No	No
Graham et al.^17^	Yes	Yes	No	No	Yes	Yes	Yes	No
Graham et al.^18^	No	No	No	No	Yes	Yes	No	No
Haug et al.^19^	No	No	No	No	Yes	Yes	Yes	No
Heffner et al.^20^	Yes	Yes	Yes	No	Yes	Yes	No	No
Jain et al.^21^	Yes	No	No	Yes	No	No	No	No
Krishnan et al.^22^	No	Yes	No	No	Yes	No	Yes	Yes
Noonan et al.^23^	Yes	No	No	No	No	No	Yes	No
Orfin et al.^24^	Yes	Yes	No	Yes	No	Yes	Yes	No
Palmer et al.^25^	Yes	No	No	No	No	No	No	Yes
Raiff et al.^26^	No	No	No	No	No	No	No	Yes
Rasool et al.^27^	Yes	Yes	Yes	No	No	No	No	No
Rigotti et al.^28^	No	No	No	No	No	Yes	No	No
Siddiqi et al.^30^	Yes	No	Yes	Yes	Yes	No	No	No
Siddiqui et al.^29^	Yes	No	No	Yes	No	No	No	No
Webb et al.^31^	No	No	No	No	No	Yes	No	No

#### Relevance (69.56%)

Provided an assessment of disease burden within the community (i.e. vaping or non-combustible nicotine prevalence rates) and identified unmet service needs (such as a lack of youth-focused service)^14^.

#### Community engagement (28.09%)

Developed interventions in partnership with product users^24^, and conducted qualitative interviews prior to intervention development to identify the needs of the community or target population^22,27^.

#### Stakeholder collaboration (13.04%)

Collaborated with community advisors with lived experience to implement the intervention^20^, and worked with stakeholders to identify appropriate recruitment strategies^27^.

#### Ethically sound (17.39%)

Dedicated time to the development of culturally sensitive study materials^30^, ensured fair distribution of support among economically disadvantaged communities^29^.

#### Replicable (60.87%)

Provided clear descriptions of study materials and procedures^11^, used expertise and resources that could be generalized to other contexts^22^, and provided intervention fidelity assessments^15^.

#### Effective (52.17%)

Reported statistically significant abstinence outcomes^17,33^ and used objective validation methods such as biochemically validated CAR^12^.

#### Efficient (21.74%)

Evidenced low requirements for study staff time and expertise to deliver the intervention^23^, and provided a full feasibility and cost analysis for the intervention^24^.

#### Sustainable (13.04%)

Provided specific instructions for ensuring continuity of the intervention^25,26^.

### What are the perceived or actual barriers and facilitators to implementation?

Five main factors that acted as barriers or facilitators to the successful implementation of included interventions were identified. They were as follows: methodological factors, participant engagement, intervention accessibility, psychosocial factors, and scalability and sustainability. An overview is provided in Table 3.

**Table 3:** Key barriers and facilitators, with examples, among 23 included vaping and non-combustible nicotine cessation interventions, identified through a systematic search of three databases of peer-reviewed journal articles (MEDLINE, PsycINFO, Scopus), published between 1 January 2003 and 18 November 2025

	As a barrier	As a facilitator
**Methodological factors**	Limits to generalizability through non-representative sampling	Use of objective abstinence measures
**Participant engagement**	Lack of sustained interaction; passive rather than active engagement	High flexibility and autonomy
**Intervention accessibility**	Messaging being too long or repetitive	Digital and remote delivery of intervention content
**Psychosocial factors**	Anxiety surrounding quitting	Social support; developing trust and rapport
**Scalability and sustainability**	Staff time and expertise required to deliver the intervention	Intervention materials that could be adapted to other settings

### Methodological factors

The use of objective abstinence measures (i.e. biochemical verification) was a consistent factor that authors of the included studies considered when evaluating the accuracy of their findings. Caponnetto et al.^12^ cited salivary cotinine-derived measures as a means of objectively verifying abstinence from vaping in their study. Where biochemical validation was not used, this was speculated to have resulted in potential recall bias due to over-reporting of abstinence rates by relying on self-report^17^. Moreover, the need for vaping-specific biochemical verification was raised^25^. This lack of objective validation served to limit the internal validity of included studies, and overall confidence in the cessation outcomes. Another methodological constraint concerned limits to the external validity of findings. This was primarily due to study samples not being representative of various groups: dual-users^15^; those less motivated to quit^20^; users of other nicotine products^16^ and adolescents^12^. These limits to generalizability could impinge the extent to which findings can be translated to real-world contexts or broader populations.

### Engagement

Participant engagement, when discussed as a barrier, mainly presented itself in terms of lower than expected levels of interaction with the intervention^15^, or challenges measuring engagement of users who passively accessed, rather than actively used, digital interventions^32^. Noonan et al.^23^ also stated that 51.9% of their participants reported the intervention timeframe in their study as being too short. Long waiting times can also result in a drop-off in engagement, as discussed by Rasool et al.^27^. By contrast, interventions that offered a degree of flexibility and autonomy were received favorably, particularly in youth-focused programs. For instance, choice and empowerment were fostered by encouraging participants to set or reset a quit date^18^.

### Accessibility

A few studies highlighted issues that were encountered in terms of poorly optimized intervention design and accessibility. These issues were mainly apparent when messages delivered during text-based programs were reported as being too long or repetitive, and thus less accessible^19^. Interventions that were designed to initiate abrupt abstinence were also found to have resulted in higher withdrawal symptoms and relapse rates^30^. In terms of high-accessibility interventions, which was the most consistently reported facilitator, this was especially prominent in studies that used text-based, app-based, or social media-based programs^15,19,23,25,32^. These programs also circumvented a need for clinic visits, which had the advantage of being able to reach members of rural or underserved communities^23^.

### Psychosocial factors

A number of psychosocial barriers were mentioned in included studies. Caponnetto et al.^12^ found that anxiety was associated with reduced odds of successful continuous abstinence, in addition to living with other vape users. By contrast, placing emphasis on positive social interactions was a strong facilitator, when social support was leveraged. For instance, the high levels of engagement observed in Do et al.^32^ were primarily attributed to the availability of social support in their online program. The use of counsellors who were matched in age with participants was also highlighted as a strength^15^. Other methods of developing trust and rapport, such as through the use of online communities^32^ were also highlighted as a means of promoting high intervention uptake.

### Scalability and sustainability

Finally, Palmer et al.^25^ observed a number of barriers related to the scalability and sustainability of their financial incentive-based intervention. This was mainly discussed in terms of the intervention requiring substantial staff time and infrastructure, as well as the cost of the incentives themselves. Feasibility constraints therefore stood out as a major concern affecting uptake and engagement with cessation interventions.

### What treatment preferences have been reported by those who vape and/or use non-combustible nicotine products?

Treatment preferences were addressed (directly or indirectly) in 20 of the 23 included studies. Overall, the available evidence pointed towards participants showing a preference for: digital and remote intervention delivery; low-burden intervention content; the availability of human coaching and support; approaches that empowered participants; and interactive over static intervention content. Text-based programs such as ‘This Is Quitting’ (TIQ) showed high satisfaction and recommendation rates^33^. Remote delivery also assisted in expanding the reach of recruitment – 43% of the Palmer et al.^25^ sample were recruited from outside the local area of their study. App-based interventions also showed continued engagement during and after the set quit date by participants^31^. Therefore, participants generally preferred cessation supports that were private, accessible, and did not require clinic attendance. Interventions that were easy to use and imposed minimal cognitive demand were also well-received. This was most evident among those which provided fast response times and on-demand cessation tips and strategies^23^. In the study of Orfin et al.^24^, 88.9% of participants reported being satisfied or extremely satisfied with the intervention, which prioritized low-effort engagement requirements. Additionally, 83.3% of their participants rated the intervention as providing ‘just the right amount’ of messages. Thus, interventions which could be integrated more seamlessly into the daily lives of participants appeared to be received favorably.

Participants also showed a preference for interventions that provided person-to-person support. The availability of quit coaches was rated as the most helpful component of their app-based intervention^31^. The presence of supportive staff was also reported as the most popular component of the intervention of Raiff et al.^26^, as well as the availability of regular video calls. Benefits of confidence-building approaches were also observed – in the work of Palmer et al.^25^, 88% of participants reported improved confidence in quitting vaping, and 80% reported feeling greater control of their vaping behaviors. The intervention of Krishnan et al.^22^, which explicitly focused on building self-efficacy, had higher program satisfaction rates than standard e-cigarette cessation informational supports (55.6% vs 17.7%, a statistically significant difference). Graham et al.^33^ also concentrated on growing ‘behavioral capability’ to quit vaping. Those assigned to the intervention reported higher program satisfaction than participants in the control group who only received text messages, and reported feeling significantly more confident in quitting. These empowerment approaches appeared to be particularly salient among youth and young adult samples^25,33^. Lastly, the use of visual, interactive content was preferred over more text-heavy approaches. In the study of Siddiqi et al.^30^, participants reported that the image-based cards used in the study were both more engaging and more motivating than other components. The authors suggested that as a consequence of using visual and interactive materials, participants had a greater understanding of the harms of non-combustible nicotine, and had greater intentions of quitting.

Finally, the literature on non-combustible nicotine cessation has provided several tangible opportunities which could be applied to enhancing the effectiveness of vaping cessation interventions. These recommendations are outlined in Supplementary file Appendix C.

## Discussion

Overall, the findings indicated that interventions for the cessation of these products showed alignment with best practice in the areas of relevance, replicability, and effectiveness, but there were shortcomings in several other key areas, such as sustainability and stakeholder collaboration. Methodological factors, participant engagement, accessibility, psychosocial factors, and scalability and sustainability were the main factors that affected the implementation of these interventions. User-reported treatment preferences indicated strong support for interventions that provided low-burden digital support, with the presence of human coaching, empowerment, with interactive content also being preferred. The findings of this review have important implications for both research and practice, which are discussed below.

One novel contribution of this rapid review, which distinguishes it from previous reviews such as a recent Cochrane review by Butler et al.^9^, is that it included interventions which targeted both vaping and non-combustible nicotine products (i.e. snus, snuff, dip). This facilitated the identification of a number of key distinctions between the two types of interventions, both in terms of their content and the barriers and facilitators that were relevant. Interventions for vaping cessation tended to draw on innovative, digitally delivered behavioral approaches with mixed outcomes, whereas non-combustible nicotine studies relied more on established pharmacological and clinician-led approaches, with more consistent cessation effects. The key barriers affecting vaping cessation studies were typically concentrated around social influences, participant engagement, and measurement challenges, whereas non-combustible nicotine studies more often discussed physiological withdrawals, and cultural or structural barriers to treatment and abstinence. Lastly, the key facilitators which aided vaping cessation interventions were mostly to do with the usability and accessibility of the intervention, whereas intrinsic motivation (i.e. readiness to quit), culturally adapted intervention content, and integration with existing healthcare systems were the main enablers to non-combustible nicotine cessation programs.

In an assessment of included interventions in terms of their alignment with best practice, using a framework by Ng and De Colombani^6^, we found that relevance to the needs and context of the population was apparent in the majority of studies. As part of this, many studies identified unmet needs or service gaps, such as a lack of cessation support in rural or underserved areas^23^, highlighting potential inequalities in access. Relevance was also evidenced through modes of delivery being tailored to target demographics. The review also found that high-accessibility interventions were both a facilitator to implementation and were endorsed by participants. Taken together, these findings demonstrate the value of prioritizing accessibility in vaping and non-combustible nicotine interventions, not only for maximizing their effectiveness but also for increasing their reach. A review of digital vaping cessation interventions by Fonteyne et al.^35^ found that mobile apps, text-based programs, and web-based platforms constituted a scalable alternative to traditional cessation approaches. This review adds to this knowledge by showing that when accessible interventions are implemented, they are well-received. Cessation approaches that offer multiple entry points, particularly for addressing inequalities in access to services, should be explored in more detail in future research.

One barrier highlighted by the studies included in this review concerned issues with the scalability and sustainability of interventions. This was echoed by the assessment of interventions’ alignment with best practice, which found that very few studies had factored this in to the implementation of their program. For a public health intervention to be considered sustainable, it should demonstrate potential continuation and have the ability to be implemented at a local level^6^. Interventions which use text-based cessation support and varenicline, where available, show considerable potential to be delivered at scale, as programs can be standardized and readily applied to other contexts. Therefore, scalable digital and hybrid intervention models represent a promising means of realizing long-term abstinence for those who seek cessation support. However, there is currently limited high-certainty evidence regarding the effectiveness of behavioral support for vaping cessation^9^. Additional randomized controlled trials and implementation studies are needed to determine which intervention components are most effective.

In order for vaping and non-combustible nicotine interventions to be applied to other contexts, however, they should be adapted to the social and environmental influences that are driving vaping and nicotine proliferation in a given context. Interventions for vaping and other forms of nicotine product cessation can be improved by directly involving users and experts in the co-design process, thus leading to a more targeted and engaging approach. Specific ways in which public and patient involvement (PPI) can be included in the intervention design phase, which were evidenced by some studies in this review, comprised interactive workshops^30^, the use of semi-structured interviews^27^, tailoring intervention content to cultural norms^24^, and using peer support-based digital programs^32^. Nicotine addiction is influenced by a complex combination of factors, and future research should implement stakeholder perspectives from the early stages through qualitative investigation or, if this is not feasible, through piloting the intervention with representatives of the population.

### Strengths and limitations

The key strengths of this review are that it has provided specific guidance on how to improve future interventions through integration of barriers and facilitators, it has evaluated the real-world implementation of these interventions rather than concentrating solely on efficacy, and it has integrated findings that concern diverse nicotine product types. These strengths also serve as novel contributions over other reviews in this area^9,35^.

The review also has a number of limitations which should be considered. Firstly, while recommendations for vaping cessation approaches have been drawn from the non-combustible nicotine studies included in the review, including these studies introduced a level of heterogeneity to the findings that made evidence synthesis challenging, particularly given the range of approaches already apparent within studies targeting the same type of product. However, it was decided that inclusion of both types of studies would more accurately reflect real-world, poly-nicotine consumption, which is characterized by repeated substitution of one product for another^4^. There are also a number of limitations inherent to conducting a rapid rather than systematic review, including greater vulnerability to bias through omitting quality appraisal, and a narrower scope reducing comprehensiveness. Finally, no interventions for the cessation of synthetic nicotine products (e.g. pouches) were identified, thus limiting the timeliness of the findings of this review. This absence is likely a consequence of the rapid pace of product development, with newer products such as nicotine pouches developing far more rapidly than the development of corresponding interventions. This raises the need for greater agility in intervention development and evaluation. It also raises the question of whether the effective components of cessation interventions are transferable across nicotine products, or whether product-specific characteristics (e.g. mode of nicotine delivery) require tailored approaches. Addressing this will be of critical importance to ensuring interventions remain relevant in a rapidly changing marketplace.

## Conclusions

This rapid review has evaluated the extent to which interventions for vaping and alternative nicotine product cessation align with best practice in a real-world setting, and has provided several concrete suggestions for improved implementation. Specifically, accessibility, sustainability, and stakeholder and community engagement appear critical to ensuring long-term success. The findings of this review can be used to inform the development and refinement of vaping and non-combustible nicotine product cessations interventions, through leveraging the factors which appear to enhance successful implementation. For such initiatives to be successful, they will need to be responsive to the needs and motivations of the target population. Standardized, evidence-based guidance will also significantly reduce the burden placed on clinicians to develop appropriate treatment strategies. However, this is a complex and rapidly evolving area, particularly given the growing use of a diverse range of nicotine products among specific cohorts such as young people. An agile response to this will be required, from both researchers and clinicians, to keep pace with changing patterns of use. One key challenge will be to determine which elements of effective interventions are generalizable across nicotine product types, and which require tailoring to specific product characteristics. Given current trajectories of use, the need for best practice interventions is unlikely to diminish.
