# Supplementary material for: International best practice in the cessation of vaping and non-combustible nicotine use, and barriers, facilitators and treatment preferences: A rapid review
Source: Tob Prev Cessat. 2026 Oct 16;12:10.18332/tpc/226396. doi: 10.18332/tpc/226396 (PMC13715562; doi:10.18332/tpc/226396)
Supplement: Supplementary file 1 [file TPC-12-65-s001.pdf]

TOBACCO PREVENTION AND CESSATION

Supplementary file

© 2026 Coyne R. et al.

DOI:
10.18332/tpc/226396

The content has been provided by the author(s) and has not been reviewed, verified, or endorsed by European Publishing. It may not have undergone peer review. The views, opinions, and recommendations expressed are solely those of the author(s) and do not necessarily reflect the position of European Publishing. European Publishing accepts no responsibility or liability for any consequences arising from the use of, or reliance on, this content.

Appendix A – Search strategies

Database: PsycINFO and PsycEXTRA (EBSCOhost)

S1

(DE "Electronic Cigarettes"

OR TI(vaping* OR vapes* OR e-cig* OR electronic cigarette* OR electronic vaping device* OR electronic nicotine delivery system* OR pod mod* OR JUUL OR heated tobacco OR heat-not-burn OR HNB OR nicotine pouch* OR oral nicotine OR snus OR smokeless tobacco OR alternative nicotine OR novel nicotine OR zyn OR freebase nicotine OR puff bar OR elf bar OR vapo?rette* OR electr* hookah* OR e-liquid OR e-juice)

OR AB(vaping* OR vapes* OR e-cig* OR electronic cigarette* OR electronic vaping device* OR electronic nicotine delivery system* OR pod mod* OR JUUL OR heated tobacco OR heat-not-burn OR HNB OR nicotine pouch* OR oral nicotine OR snus OR smokeless tobacco OR alternative nicotine OR novel nicotine OR zyn OR freebase nicotine OR puff bar OR elf bar OR vapo?rette* OR electr* hookah* OR e-liquid OR e-juice))

NOT

(DE "Tobacco Smoking"))

S2

(DE "Harm Reduction" OR DE "Counseling" OR DE "Psychotherapy" OR DE "Treatment OR DE "Mobile Applications" OR DE "Telemedicine" OR DE "Motivational Interviewing" OR DE "Cognitive Behavior Therapy"

OR TI(cessation OR quit* OR end OR stop* OR discontinue OR harm reduction OR relapse prevention OR withdrawal management OR counsel* OR psychotherap* OR treatment* OR intervention* OR quitline* OR mobile app* OR mHealth OR eHealth OR motivational interview* OR trial* OR cognitive behavio* therapy OR CBT OR financial incentive*)

OR AB(cessation OR quit* OR end OR stop* OR discontinue OR harm reduction OR relapse prevention OR withdrawal management OR counsel* OR psychotherap* OR treatment* OR intervention* OR quitline* OR mobile app* OR mHealth OR eHealth OR motivational interview* OR trial* OR cognitive behavio* therapy OR CBT OR financial incentive*))

S3

(DE "Client Attitudes" OR DE "Consumer Measures" OR DE "Attitudes" OR DE "Perception" OR DE "Treatment Compliance"

OR TI(preference* OR accept* OR attitud* OR perception* OR experience* OR barrier* OR facilitator* OR challenge* OR obstacle* OR support* OR implementation* OR uptake OR adopt* OR feasibility OR engage* OR adherence OR compliance)

OR AB(preference* OR accept* OR attitud* OR perception* OR experience* OR barrier* OR facilitator* OR challenge* OR obstacle* OR support* OR implementation* OR uptake OR adopt* OR feasibility OR engage* OR adherence OR compliance))

S4: S1 AND S2 AND S3 (Limiters: Publication Date: 20030101-[date of search]; Publication Type: Peer Reviewed Journal [PsycINFO only]; English language

Database: Medline (Ovid)

1 Vaping/

2 vaping*.ti,ab.

3 vapes*.ti,ab.

4 e-cig*.ti,ab.

5 electronic cigarette*.ti,ab.

6 electronic vaping device*.ti,ab.

7 electronic nicotine delivery system*.ti,ab.

8 Electronic Nicotine Delivery Systems/

9 pod mod*.ti,ab.

10 JUUL.ti,ab.

11 heated tobacco.ti,ab.

12 heat-not-burn.ti,ab.

13 HNB.ti,ab.

14 nicotine pouch*.ti,ab.

15 oral nicotine.ti,ab.

16 snus.ti,ab.

17 smokeless tobacco.ti,ab.

18 alternative nicotine.ti,ab.

19 novel nicotine.ti,ab.

20 zyn.ti,ab.

21 freebase nicotine.ti,ab.

22 puff bar.ti,ab.

23 elf bar.ti,ab.

24 vapo?rette*.ti,ab.

25 electr* hookah*.ti,ab.

26 e-liquid.ti,ab.

27 e-juice.ti,ab.

28 Smoking/

29 (1 OR 2 OR 3 OR 4 OR 5 OR 6 OR 7 OR 8 OR 9 OR 10 OR 11 OR 12 OR 13 OR 14 OR 15 OR 16 OR 17 OR 18 OR 19 OR 20 OR 21 OR 22 OR 23 OR 24 OR 25 OR 26 OR 27) NOT (28)

30 cessation.ti,ab.

31 quit*.ti,ab.

32 end.ti,ab.

33 stop*.ti,ab.

34 discontinue.ti,ab.

35 harm reduction.ti,ab.

36 Harm Reduction/

37 relapse prevention.ti,ab.

38 withdrawal management.ti,ab.

39 counsel*.ti,ab.

40 Counseling/

41 psychotherap*.ti,ab.

42 Psychotherapy/

43 treatment*.ti,ab.

44 intervention*.ti,ab.

45 Psychosocial intervention/

46 quitline*.ti,ab.

47 mobile app*.ti,ab.

48 Mobile Applications/

49 mHealth.ti,ab.

50 eHealth.ti,ab.

51 Telemedicine/

52 motivational interview*.ti,ab.

53 Motivational Interviewing/

54 trial*.ti,ab.

55 cognitive behavio* therapy.ti,ab.

56 Cognitive Behavioral Therapy/

57 CBT.ti,ab.

58 financial incentive*.ti,ab.

59 32 OR 33 OR 34 OR 35 OR 36 OR 37 OR 38 OR 39 OR 40 OR 41 OR 42 OR 43 OR 44 OR 45 OR 46 OR 47 OR 48 OR 49 OR 50 OR 51 OR 52 OR 53 OR 54 OR 55 OR 56 OR 57 OR 58 OR 58

60 preference*.ti,ab.

61 Patient Preference/

62 accept*.ti,ab.

63 Patient Acceptance of Health Care/

64 attitud*.ti,ab.

65 Attitude/

66 perception*.ti,ab.

67 Perception/

68 experience*.ti,ab.

69 barrier*.ti,ab.

70 facilitator*.ti,ab.

71 challenge*.ti,ab.

72 obstacle*.ti,ab.

73 support*.ti,ab.

74 implementation*.ti,ab.

75 uptake.ti,ab.

76 adopt*.ti,ab.

77 feasibility.ti,ab.

78 Feasibility Studies/

79 engage*.ti,ab.

80 adherence.ti,ab.

81 compliance.ti,ab.

82 Patient Compliance/

83 62 OR 63 OR 64 OR 65 OR 66 OR 67 OR 68 OR 69 OR 70 OR 71 OR 72 OR 73 OR 73 OR 74 OR 75 OR 76 OR 77 OR 78 OR 79 OR 80 OR 81 OR 82

84 29 AND 59 AND 83

85 limit 84 to (English language and yr=”2003-Current”)

86 limit 85 to journal article

Database: Scopus (Elseiver)

(

(TITLE-ABS-KEY (vaping* OR vapes* OR e-cig* OR "electronic cigarette*" OR "electronic vaping device*" OR "electronic nicotine delivery system*" OR "pod mod*" OR JUUL OR "heated tobacco" OR "heat-not-burn" OR HNB OR "nicotine pouch*" OR "oral
nicotine" OR snus OR "smokeless tobacco" OR "alternative nicotine" OR "novel
nicotine" OR zyn OR "freebase nicotine" OR "puff bar" OR "elf bar" OR vapo?rette* OR "electr* hookah*" OR "e-liquid" OR "e-juice")) AND NOT (KEY (smoking))

)

AND

TITLE-ABS-KEY (

cessation OR quit* OR end OR stop* OR discontinue OR "harm reduction" OR "relapse prevention" OR "withdrawal
management" OR counsel* OR psychotherap* OR treatment* OR intervention* OR quitline* OR "mo
bile app*" OR mHealth OR eHealth OR "motivational interview*" OR trial* OR "cognitive behavio* therapy" OR CBT OR "financial incentive*"

)

AND

TITLE-ABS-KEY (

preference* OR accept* OR attitud* OR perception* OR experience* OR barrier* OR facilitator* OR challenge* OR obstacle* OR support* OR implementation* OR uptake OR adopt* OR feasibility OR e
ngage* OR adherence OR compliance

)

AND

(PUBYEAR > 2002) AND (LIMIT-TO (DOCTYPE , "ar")) AND (LIMIT-TO (LANGUAGE , "English"))

Appendix B. Characteristics of N = 23 included vaping and non-combustible nicotine cessation interventions identified through a systematic search of three databases of peer-reviewed journal articles (MEDLINE, PsycINFO, Scopus), published between January 1st, 2003 and November 18th, 2025

Type of product(s) used	Study	*N*	Age *M* (*SD*)	Gender % M / % F	Study type (Randomised/non-randomised); Intervention type	Frequency of intervention	Modality of intervention	Duration of intervention	Results
Vapes	Caponnetto et al., 2023	140	52.6 (9.1)	49.29% M 50.71% F	Non-randomised; Pharmacological (varenicline) and behavioural (cessation counselling)	Weekly for 12 weeks; follow-up at 24 weeks	Over-the-counter varenicline and in-person counselling	12 weeks	CAR between weeks 4-12 and 4-24 significantly higher for varenicline versus placebo at weeks 4-12 (40% vs 20%, *p* = 0.011) and weeks 4-24 (34.3% vs 17.2%, *p* = 0.022)<br>7-day PPA at weeks 12 and 24: Also higher for varenicline than placebo at each time point (41.4% vs 22.9%, *p* = 0.02 at week 4, and 34.3% vs 17.2%, *p* = 0.022 at week 24).
Vapes	Do et al., 2024	290	13-24 years	28% M 67% F 4% NB	Non-randomised; Behavioural (interactive text-based intervention)	Daily, with follow-up at 1 month	Text-based program with a social media platform (Discord)	1 months	Abstinence at follow-up: Engaging with TIQ Discord was positively associated with increased confidence in quitting at follow-up (*p* = 0.02), but vaping abstinence at follow-up did not differ (*p* = 0.87).
Vapes	Evins et al., 2025	254	21.4	47% M 53% F	Randomised; Pharmacological (varenicline) and behavioural	Weekly for 12 weeks; follow-up at weeks 16, 20 and 24	In-person and online; varenicline distributed via hospitals	12 weeks	CAR for the varenicline plus behavioural counselling and placebo plus behavioural

					(cessation counselling)				counselling groups were 51% vs 14%, p < .001 over the last 4 weeks of treatment (weeks 9-12) and 28% vs 7%, p < .001 over weeks 9 through 24. Those in the varenicline plus behavioural counselling group had higher CAR than those in the enhanced usual care group over weeks 9 through 12 (51% vs 6%, $p < .001$) and over weeks 9 through 24 (28% vs 4%, $p = 0.001$).
Vapes	Graham et al., 2021	2588	20.4 (1.7)	48.4% M<br>50.3% F<br>1.3% NB	Non-randomised; Behavioural (interactive text-based intervention)	Weekly, for between 4 and 8 weeks; follow-up at 1 and 7 months	Text-based program	Up to 8 weeks	30-day PPA rates at 7 months were 24.1% among TIQ participants and 18.6% among controls, a statistically significance difference ($p < .001$).
Vapes	Graham et al., 2024; 2025 (follow-up secondary analysis)	1503<br>;<br>1248	16.4 (0.8)	41% M<br>50% F<br>9% NB	Non-randomised; Behavioural (interactive text-based intervention)	Weekly, for between 4 and 8 weeks; follow-up at 1 and 7 months	Text-based program	Up to 8 weeks	30-day PPA at 7 months: 37.8% among intervention participants and 28.0% among assessment-only participants ($p < .001$).
Vapes	Haug et al., 2025	167	23.2 (4.0)	43.1% M<br>56.9% F	Non-randomised; Behavioural (text-based coaching)	Continuous monitoring over 10 weeks	Text-based program	10 weeks	Number of days using electronic nicotine products in the last 30 days was M = 11.1 at baseline and M = 7.7 at follow-up; a statistically significant decrease ($p = 0.005$).

Vapes	Heffner et al., 2025	61	21.8 (3.4)	42.6% M<br>57.4% F	Randomised; Behavioural (acceptance and commitment therapy and text-based support)	Continuous monitoring over 12 weeks	Text-based program	12 weeks	24-hour quit attempts: 87.5% in the ACT on Vaping arm versus 75.9% in control. Cotinine-confirmed 30-day PPA was 4.2% in ACT arm vs 0% in control.
Vapes	Krishnan et al., 2023	58	27.3 (5.5)	53.5% M<br>46.5% F	Non-randomised; Behavioural (text-based support)	Weekly; daily messages for first 2 weeks	Text-based program	4 weeks	QTV-C participants (vs QTV and control) trended more favorably on 7-day e-cigarette PPA [27.8% vs 11.1% and 5.9%, *p* = 0.165], and quit attempts [66.7% vs 50.0% 52.9%, *p* = 0.561] but differences not statistically significant
Vapes	Orfin et al., 2024	40	22.3 (2.1)	47.5% M<br>45% F<br>7.5% NB	Non-randomised; Behavioural (text-based support)	212 messages delivered over 3 months	Text-based program	3 months	75% of participants self-reported 7-day point prevalence abstinence.
Vapes	Palmer et al., 2022	27	20.3 (1.2)	33.3% M<br>66.7% F	Non-randomised; Financial incentive-based and behavioural (text-based support)	Every 2-3 days over 4 weeks	Smartphone app and text-message service	4 weeks	No differences in abstinence between groups at EOT (*p* = 0.87) or follow-up (*p* = 0.44).
Vapes	Raiff et al., 2022	8	19.88	37% M<br>63% F	Non-randomised; Financial incentive-based	Every other day over 2 weeks	Online; synchronous video calls	2 weeks	Biochemically-verified cessation: Sixteen percent of samples (n = 9) met the criteria of “no nicotine exposure” (Level 0), 9% of the samples (n = 5) were Level 2, 4% (n = 2) were Level 4, and 2% (n = 1) were Level 3.

Vapes	Rigotti et al., 2024	160	33.6 (11.1)	48.1% M 51.9% F	Randomised; Pharmacological (cytisinicline) and behavioural (cessation counselling)	Daily cytisinicline with weekly behavioural support	In-person clinic visits	12 weeks	CAR higher in cytisinicline condition vs placebo (31.8% vs 15.1%, *p* = 0.04) at end of treatment (weeks 9-12). No significant difference during weeks 9 to 16 (23.4% vs 13.2%, *p* = 0.15).
Vapes	Webb et al., 2023	51	27.9 (8.1)	45.4 % M 54.6% F	Non-randomised; Behavioural (mobile intervention combining self-guided CBT and virtual coaching) and pharmacological (NRT)	30-minute call with quit coach; continuous app access; 6 weeks of NRT	Mobile app	6 weeks	At 1-month post-quit date, 48.9% (22/45) of study completers reported 7-day PPA and 28.8% (13/45) reported continuous 30-day abstinence.
Non-combustible nicotine (chew and snuff)	Boyle et al., 2008	406	39.9 (10.9)	98.3% M 1.7% F	Randomised; Behavioural (Informational resources, telephone counselling)	Weekly calls as needed to stay quit; follow-up at 3 and 6 months	Telephone-based program	Up to 4 treatment calls	Participants randomised to the telephone counselling group reported significantly higher prolonged and PPA abstinence at both follow-up points (30.9% vs 6.8%, *p* < .001). Prolonged abstinence from tobacco was more likely for participants if they were in the telephone counselling group.
Non-combustible nicotine (chew).	Danaher et al., 2013	1716	20.8 (2.64)	96.5% M 3.5% F	Non-randomised; Behavioural (informational resources, interactive media)	Once-off webpage with follow-up at 3 and 6 months	Web-based program	Once-off web-based program (11 pages)	No significant differences between the Enhanced Condition and the Basic Condition. Participants who continued to use smokeless tobacco (ST) at both 3- and

									6-month assessments points reported that cans of ST lasted more days compared with use at baseline (*M* = 2.04 vs 3.12, *p* < .001) and 6-month assessment (*M* = 3.51, *p* < .001). Additionally, these participants reported making more quit attempts at 3 months compared with baseline (*M* = 2.17 vs 2.90, *p* < .001) and at 6 months (*M* = 2.92, *p* < .001).
Non-combustible nicotine	Ebbert et al., 2010	60	43.6 (16.0)	100% M	Non-randomised; Pharmacological (nicotine lozenges) and behavioural (self-help guides)	Daily for 12 weeks	Mailed medication with contact over the phone	12 weeks	At 12 weeks, 7-day PPA was 47% in the nicotine lozenge group and 37% in placebo (*p* = 0.432), while prolonged abstinence was 43% vs. 37% (*p* = 0.598). At 6 months, PPA was 33% in the nicotine lozenge group and 47% in placebo (*p* = 0.184), and prolonged abstinence rates were 27% and 38% (*p* = 0.405), respectively.
Non-combustible nicotine (snus)	Fagerstrom et al., 2010	431	43.9 (12.0)	64% M 36% F	Randomised; Pharmacological (varenicline) and behavioural (brief counselling)	Daily for 12 weeks; follow-up from weeks 9-12 and at week 26	In-person clinic visits	26 weeks	CAR at weeks 9-12 was significantly higher in the varenicline group than in the placebo group (59% vs 39%, *p* < .001). CAR also significantly higher in the long term (weeks 9-26) in the

									varenicline group than in the placebo group (45% vs 34%, *p* = 0.012). 7-day PPA was also higher at week 12 in the varenicline group than the placebo group (58% vs 39%, *p* < .001).
Non-combustible nicotine	Jain et al., 2014	237	34.2 (9.2)	97% M 3% F	Non-randomised; Pharmacological (varenicline) and behavioural (cessation counselling)	Daily medication used; biweekly counselling	In-person counselling and over-the-counter varenicline	12 weeks	Abstinence was greater for varenicline versus placebo (25.2% vs. 19.5%), but there was no statistically significant difference (*p* = 0.15). Compared with placebo, varenicline did not reduce the risk for a lapse, but it did increase the likelihood of recovery to abstinence.
Non-combustible nicotine (snuff)	Noonan et al., 2024	532	44.9 (11.9)	99.3% M 0.7% F	Non-randomised; Behavioural (text-based support)	Daily for 10 weeks via automated text mesages	Text-based program	10 weeks	3-month abstinence higher in intervention 29.2% than Control 19.0%, *p* = 0.007). No significant differences at 6-month abstinence (*p* = .5384). Reduction among non-quitters at 3 months was 57.1% for intervention, and 50% for control (*p* = .0492).
Non-combustible nicotine (dip)	Rasool et al., 2024	100	41.3 (17.0)	99% M 1% F	Non-randomised; Behavioural (cessation counselling)	Visits over a 5–6-week period with follow-up at 6 months	In-person sessions with dentists	5-6 weeks	Self-reported abstinence at Visit 3: was at 12% in the control group, and 38% in the intervention group. At 3 months, abstinence was 8% in the control group and

									34% in the intervention group. Self-reported 6-month abstinence was 8% for the control group at 24% for the intervention group. Biochemically verified abstinence was 10% for the intervention group and 4% for controls.
Non-combustible nicotine	Siddiqi et al., 2016	32	Not reported	50% M 50% F	Non-randomised; Behavioural (educational resources)	Three sessions between 4 and 54 minutes; pre-quit, quit, and post-quit; follow up at 6 months	In-person counselling	6 months	12.5% of participants demonstrated biochemically verified abstinence at 6 months.
Non-combustible nicotine	Siddiqui et al, 2023	264	35.0 (12.5)	53% M 47% F	Randomised; Behavioural (self-help resources) and pharmacological (NRT)	Weekly sessions up to six weeks; follow-up at 6, 12 and 26 weeks	In-person and telephone-based sessions	26 weeks	Self-reported abstinence was at 65.2% at 6 weeks, and 55.7 at 12 and 26 weeks. Only 8.7% had biochemically verified abstinence.

ACT = Acceptance commitment therapy
CAR = Continuous abstinence rate
EOT = End of treatment
F = Female
SD = Standard deviation
M (Gender) = Mean
M (Age and Results) = Male
NB = Non-binary
NRT = Nicotine replacement therapy
PPA = Point prevalence abstinence
QTV-C = Quit the vape with counsellor-delivered messages
QTV = Quit the vape
ST = Smokeless tobacco
TIQ = This Is Quitting

Appendix C. Recommendations for developing vaping cessation interventions based on insights garnered from N = 23 included studies identified through a systematic search of three databases of peer-reviewed journal articles (MEDLINE, PsycINFO, Scopus), published between January 1st, 2003 and November 18th, 2025

**Recommendation**	Insight from non-combustible nicotine literature	Application to vaping
**1. Cultivate motivation to quit**	Many non-combustible nicotine users enrol in interventions with existing high readiness to quit, and interventions emphasise health concerns, financial incentives, and culturally relevant influences.	Vaping cessation approaches should capitalise on non-combustible nicotine interventions' motivational strategies, to support vape users maintaining strong intentions to quit.
**2. Treat withdrawal and dependence more seriously**	Non-combustible nicotine users frequently report withdrawals, and interventions explicitly plan for nicotine substitution and gradual reduction.	Vaping cessation interventions should frame vaping more clearly as a form of nicotine dependence and implement more structured withdrawal management approaches.
**3. Embed cessation programs into existing health systems**	Non-combustible nicotine studies are embedded in widely accessible, existing healthcare systems, reducing the need for sustained engagement with lone programs.	Many vaping cessation interventions exist as standalone programs; digital supports may benefit from being integrated in primary care units, student health centres, or pharmacies.
**4. Implement pharmacotherapy as a core intervention component**	Pharmacotherapy (varenicline, NRT) is	Vaping cessation should more explicitly adopt

	effective in terms of quitting non-combustible nicotine, as well as recovery after relapse	pharmacological approaches; behavioural-only approaches are not an effective replacement for medication.
**5. Standardise outcome measures and verification**	Non-combustible nicotine studies rely on a more standardised set of outcome measures, improving the interpretability and credibility of findings.	Vaping cessation studies use heterogenous outcomes (7-day PPA, 30-day PPA) – using outcomes more aligned with non-combustible nicotine literature would facilitate better distinction of true cessation rates from temporary gains or social desirability bias.
